# The Rice *Xa3* Gene Confers Resistance to *Xanthomonas oryzae* pv. *oryzae* in the Model Rice Kitaake Genetic Background

**DOI:** 10.3389/fpls.2020.00049

**Published:** 2020-02-06

**Authors:** Furong Liu, Weiguo Zhang, Benjamin Schwessinger, Tong Wei, Deling Ruan, Pamela Ronald

**Affiliations:** ^1^ Department of Plant Pathology and the Genome Center, University of California, Davis, Davis, CA, United States; ^2^ Faculty of Life Science, Northwest University, Xi’an, China; ^3^ Research School of Biology, Australian National University, Canberra ACT, Australia; ^4^ Joint BioEnergy Institute, Emeryville, CA, United States

**Keywords:** rice, rice bacterial blight, XA21, XA3, *Xanthomonas oryzae* pv. *oryzae*

## Abstract

The rice XA21 and XA3 pattern receptor kinases, derived from *Oryza longistaminata* and an *Oryza. sativa japonica* cultivar Wase Aikoku 3, respectively, confer resistance to strains of the Gram-negative bacterium *Xanthomonas oryzae* pv. *oryzae* (*Xoo*), the causal agent of rice bacterial blight disease. Previously, we showed that transfer of *Xa21* to the model rice cultivar Kitaake enhances resistance to *Xoo*. In this manuscript we demonstrate that Kitaake expressing *Xa3* confers resistance to *Xoo* strain PXO79 and that the stress-related marker genes *PR10b* and *KO5* are upregulated in *Xoo*-infected *Xa3* rice leaves. We also show that rice somatic embryogenesis receptor kinase 2 (OsSERK2) positively regulates XA3-mediated immunity in Kitaake. We found that overexpression of XA21 binding protein 15 (XB15) and XB24, two negative regulators of XA21-mediated immunity, do not affect XA3-mediated immunity in the Kitaake genetic background. Our results indicate that the rice immune receptors XA21 and XA3 employ both shared and distinct signaling components in their response to *Xoo*. The results are important to further understand pathogen-associated molecular pattern (PAMP)-triggered immunity in rice. Furthermore, the presence of Kitaake rice carrying *Xa3* will facilitate genetic research to study the XA3-mediated immunity.

## Introduction

Plants detect and defend against diverse microbes *via* the innate immune system (Chisholm et al., 2006). One branch of plant innate immunity is mediated by receptors localized on the cell membrane that activate the immune responses upon recognition of extracellular signals derived from pathogens (Boller and Felix, 2009). In rice, the *Xanthomonas* resistance 21 (XA21) and XA3 (also named XA26) receptor kinases confer robust resistance to strains of the Gram-negative bacterium *Xanthomonas oryzae* pv. *oryzae* (*Xoo*) that causes bacterial blight disease of rice (Song et al., 1995; Sun et al., 2004; Xiang et al., 2006). XA21 and XA3 belong to the XII subfamily of leucine-rich repeat receptor-like kinases (LRR-RLKs) and share common features typical of LRR-RLK proteins: an extracellular leucine-rich repeat domain, a transmembrane domain, and a cytoplasmic non-arginine-aspartate (non-RD) kinase domain. XA21 and XA3 share 53% amino acid sequence identity (Sun et al., 2004). XA21 contains 23 leucine-rich repeats, and XA3 harbors 26 repeats (Song et al., 1995; Sun et al., 2004). XA21 recognizes a tyrosine-sulfated protein derived from *Xoo*; the ligand for XA3 remains unknown (Pruitt et al., 2015; Luu et al., 2019). Like XA21, XA3 confers broad-spectrum resistance to most *Xoo* strains including PXO79 but not PXO99 (Song et al., 1995; Sun et al., 2004).

Based on the structural similarity of XA21 and XA3, we hypothesized that XA21 and XA3 might share components that transduce the immune response. Previous genetic studies revealed that OsSERK2 (rice somatic embryogenesis receptor kinase 2) is required for both XA21 and XA3-mediated immunity (Chen et al., 2014). In addition to OsSERK2, several other regulators of XA21-mediated immunity were previously identified and might also be involved in XA3-mediated immunity. XA21 binding protein 24 (XB24) physically associates with the XA21 juxtamembrane (JM) domain and catalyzes the autophosphorylation of XA21 at serine and threonine residue(s), keeping it in an inactive state (Chen et al., 2010b). Upon pathogen recognition, XA21 kinase dissociates from XB24 and becomes active, resulting in a robust resistance response (Chen et al., 2010b). XB15 encodes a protein phosphatase 2C (PP2C), which dephosphorylates XA21, attenuating XA21 signaling (Park et al., 2008).

*Xa21* was introgressed into diverse genetic backgrounds of cultivated rice (*Oryza sativa*) from the wild species *Oryza longistaminata* (Ikeda et al., 1990; Khush et al., 1990; Park et al., 2008). For example, *Xa21* expression in the *O. sativa* ssp. *Japonica* variety Kitaake confers robust resistance to *Xoo* strain PXO99. Kitaake has many advantages for rice genetic studies. For example, Kitaake is smaller in stature and has a much shorter life cycle (9–10 weeks) than other commonly studied rice cultivars (2–3 generations per year), allowing researchers to grow four to five generations each year (Jung et al., 2008). Kitaake is easy to propagate and is less sensitive to light quality, intensity, and photoperiod change (Jung et al., 2008). Moreover, Kitaake is highly amenable to Agrobacterium-mediated transformation (Toki, 1997), the complete sequence of Kitaake is available (https://phytozome.jgi.doe.gov/pz/portal.html#!info?alias=Org_OsativaKitaake_er) and a large collection of whole-genome sequenced Kitaake mutants is assembled (Li et al., 2017). These advantages make Kitaake an excellent model for rice genetic research. Here I have used genetic analysis to determine if XA3 confers race-specific resistance in the Kitaake genetic background and if XB24 and XB15 are required for XA3-mediated immunity.

## Materials and Methods

### Plasmid Construction and Rice Transformation

The *Xa3* coding sequence (3,312 nt) from National Center for Biotechnology Information (https://www.ncbi.nlm.nih.gov/) with or without tags was cloned into the pENTR/d TOPO vector (Chern et al., 2005). The genes were then introduced into the Gateway-compatible vector pCAMBIA4300 which contains a maize ubiquitin promoter. The constructs were transferred into the *Agrobacterium tumefaciens* strain EHA105 by electroporation. Regenerated plants were selected on mannose. The presence of the transgene was confirmed by PCR using primers which anneal to the *Xa3* sequence and the *nos* terminator in the vector (*Xa3*/F (5'-GGCAGTGGGTTCAACAGGCGT-3') and *Nos*/R (5'- AATCATCGCAAGACCGGCAACAGG-3').

*OsXb24* overexpression (A109-6-5-1) (Chen et al., 2010b), *OsXb15* overexpression (19A-72-4) (Park et al., 2008) and *OsSerk2 RNAi* (X-B-4-2) (Chen et al., 2014) transgenic plants in the Kitaake genetic background were used for crossing with *Ubi-Xa3-8-3-2* to obtain *Xa3OsXb24OE*, *Xa3OsXb15OE*, and *Xa3OsSerk2 Ri* plants. The crosses were performed using *Ubi : Xa3-8-3-2* as the pollen donor. PCR-based genotyping of *Xa3*, *OsXb24OE*, *OsXb15OE*, and *OsSerk2Ri* was performed as described previously (Park et al., 2008; Chen et al., 2010b; Chen et al., 2014). Successful crosses of *Xa3Xb24OE* and *Xa3Xb15OE* were confirmed in the F1 generation. Double transgenic plants were analyzed in the F2 generation by PCR reactions using a forward primer annealing to the *Ubiquitin* promoter and a gene-specific reverse primer (*Ubi*/F(5'-TTGTCGATGCTCACCCTGTGTTT-3'), *Xb15*/R(5'-ATGCTCTGGTCACCTTCAGCG-3') and *Xb24*/R(5'-TTACACATCTGTAATCTTGCTGC-3'). *Xa3Serk2Ri* plants were genotyped with primers annealing to the hygromycin gene in the vector (*Hyg3*(5'-TCCACTATCGGCGAGTACTTCTACACA-3') and *Hyg4*(5'-CACTGGCAAACTGTGATGGACGAC)).

### Bacterial Strains, Pathogen Inoculation, and Disease Scoring

For rice inoculation, *Xoo* strain PXO79 was grown on PSA plates [10 g of peptone (bacto-Peptone), 10 g of sucrose, 1 g of sodium glutamate (glutamic acid, monosodium salt), 16 g of bacto-agar, final volume 1L (pH: 7)] at 28°C in the dark for 2–3 days. The bacteria were resuspended in water, and the inoculum was adjusted to an optical density (O.D.) 600 of 0.5 (~5 ×10^8^ CFU/ml). Rice plants were grown in the greenhouse for five weeks and then moved into the controlled growth chamber for inoculation by the leaf-clipping method (Song et al., 1995). The temperature was maintained at 28°C with a 12-h photoperiod. Disease lesions were scored by measuring the lesion length at 14 days after inoculation.

### Bacterial Treatments of Detached Rice Leaves

Bacteria treatment of detached rice leaves was performed from 4-week old rice. Expanded adult leaves were cut into 1-cm sections using surgical-grade scissors. Samples were placed into 6-well Costar cell culture plates containing 1.5 ml of 10 mM MgCl2 solution for mock treatment or 10 mM MgCl2 containing a fresh *Xoo* cell suspension at O.D. 600 = 0.1. The plates were incubated under constant light [between 5 and 10 μmol/(m2*s)]; samples were collected 24 h post treatment for total RNA extraction (Thomas et al., 2016).

### RNA Isolation and qPCR Gene Expression Analysis of Infected Leaf Samples

Detached leaves were frozen in liquid nitrogen and disrupted with a Qiagen Tissue Lyser. RNA was extracted using the Spectrum Plant Total RNA Kit (Sigma-Aldrich). The TURBO DNase Kit (Life Technologies) was used to digest 2 μg of total RNA that was synthesized with a High Capacity cDNA Reverse Transcription Kit (Life Technologies). The ΔΔCt method was used to determine gene expression changes normalized to *Actin* (LOC_Os03g50885) and compared to mock-treated samples (Schmittgen and Livak, 2008). qRT-PCR primer pairs used in the experiments were as follows: *qActin*-F/R(5′-ATCCTTGTATGCTAGCGGTCGA-3′/5′-ATCCAACCGGAGGATAGCATG-3′), *PR10b*-F/R(5′-TGTGGAAGGTCTGCTTGGAC-3′/5′-CCTTTAGCACGTGAGTTGCG-3′) and *KO5*-F/R(5′-GCTGGCTTCCAAACAAGAGC-3′/5′-GCCTCTTGATCAACGCGTTC-3′). The qRT-PCR reaction was run for 40 cycles with annealing and amplification at 58°C for 5 s and denaturation at 95°C for 5 s.

### Protein Extraction and Western Blot Assays

Total protein was extracted from 100 mg of rice leaf tissue. The leaf sample was frozen in liquid nitrogen and disrupted with a Qiagen Tissue Lyser. Two hundreds microliter of pre-chilled extraction buffer (0.15 M NaCl, 0.01 M sodium phosphate buffer pH = 7.2, 2 mM ethylenediaminetetraacetic acid, 1% Triton X-100, 10 mM dithiothreitol, 20 mM sodium fluoride, 1 mM phenylmethylsulfonyl fluoride, 1% Sigma protease cocktail) was added, and protein was separated on an 8% sodium dodecyl sulfate-polyacrylamide gel. Flag-tagged XA3 was detected by western blot using a mouse anti-Flag primary antibody (Invitrogen) and anti-mouse IgG coupled to HRP (Santa Cruz) as a secondary antibody.

## Results

### Generation of Functional XA3 Lines in the Kitaake Rice Background

**Figure 1:**
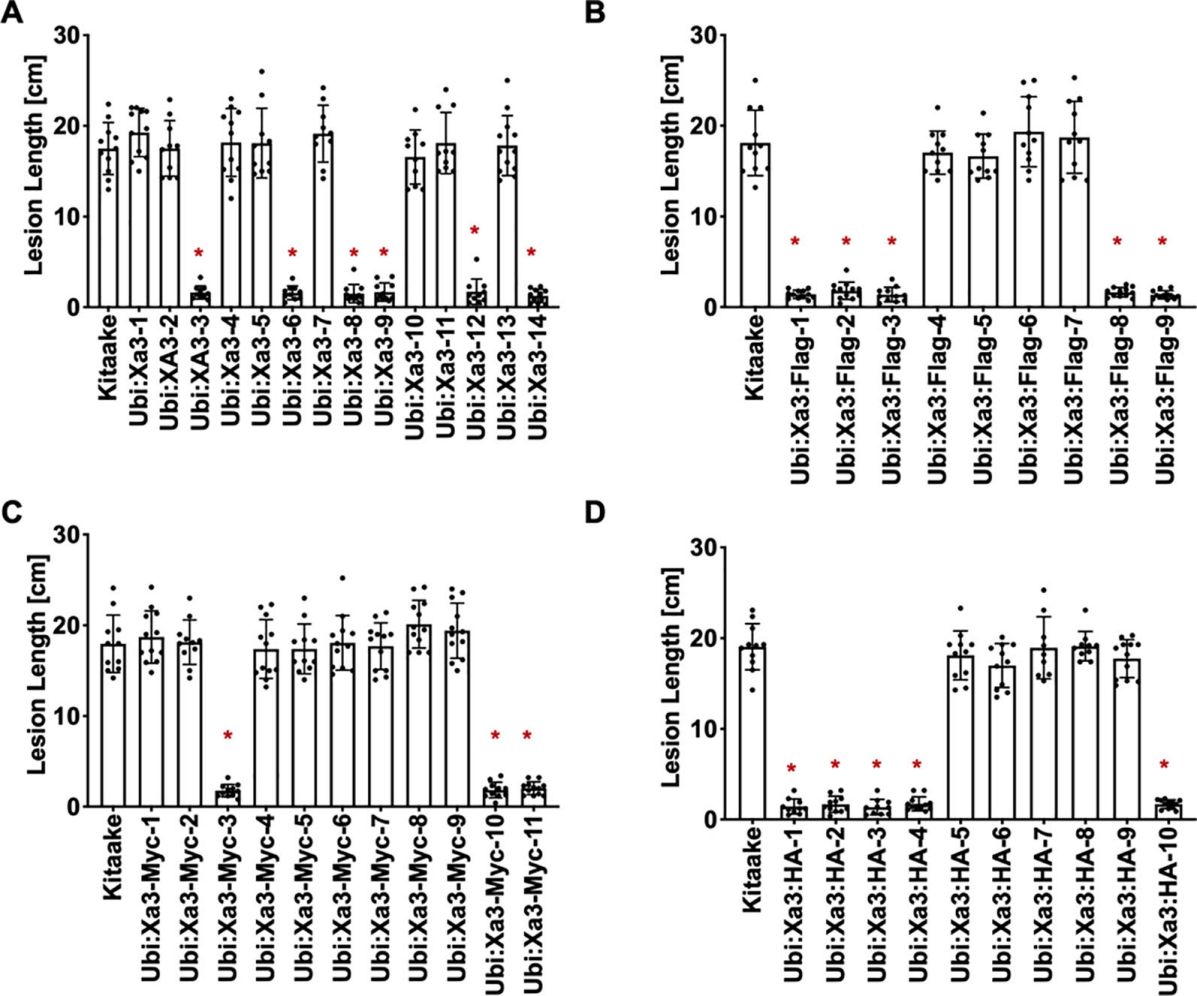
*Xa3* transgenic rice displays resistance to *Xoo* strain PXO79. The standard clipping method was used to inoculate 5-week-old T0 plants with PXO79. Lesion lengths were measured 14 days after inoculation. Bars represent mean lesion lengths for 8–12 leaves. “*” indicates a statistically significant difference from Kitaake using Dunnett’s test (α = 0.05). **(A)** Lesion length data for fourteen *Ubi : Xa3* T0 plants. Kitaake was susceptible to PXO79, and 6 out of the 14 independent lines were resistant to PXO79. **(B)** Lesion length data for nine *Ubi : Xa3:*Flag T0 plants. Five independently transformed lines were resistant to PXO79. **(C)** Lesion length data for eleven *Ubi : Xa3*:Myc T0 plants. Three independently transformed lines were resistant to PXO79. **(D)** Lesion length data for 10 *Ubi : Xa3:*HA T0 plants. Five independently transformed lines were resistant to PXO79.

XA3 was derived from *Oryza sativa japonica* cultivar Wase Aikoku 3 and confers robust resistance to *Xoo* strain PXO79 (Ezuka, 1975; Ogawa, 1987). *Xa26* was identified in Indica rice cultivar Minghui 63 and later genetic analysis showed that *Xa3* and *Xa26* are the same gene (Yang et al., 2003; Xiang et al., 2006). Here we use *Xa3* to represent the gene. To assess the function of *Xa3* in the Kitaake genetic background, the maize ubiquitin promoter was used to overexpress the *Xa3* cDNA. Fourteen independently transformed lines were generated (designated *Ubi : Xa3-1* to *Ubi : Xa3-14*). Six of these independently transformed lines showed resistance upon inoculation with PXO79 (**Figure 1A**). We also produced *Ubi : Xa3* transgenic rice lines with carboxyl-terminal tags of Flag, Myc or HA (hemagglutinin). Nine independent T0 *Ubi : Xa3:*Flag (designated *Ubi : Xa3:*Flag*-1* to *Ubi : Xa3:*Flag*-9*) (**Figure 1B**), eleven T0 *Ubi : Xa3:*Myc (designated *Ubi : Xa3:*Myc*-1* to *Ubi : Xa3:*Myc*-11*) (**Figure 1C**) and 10 T0 *Ubi : Xa3:*HA (designated *Ubi : Xa3:*HA*-1* to *Ubi : Xa3:*HA*-10*) lines were generated (**Figure 1D**). All plants were inoculated at five weeks along with the Kitaake control; lesion lengths were measured 14 days post inoculation (dpi). Five *Ubi : Xa3:*Flag, three *Ubi : Xa3:*Myc, and five *Ubi : Xa3:*HA lines showed clear resistance to PXO79 (**Figures 1B**–**D**).

**Figure 2:**
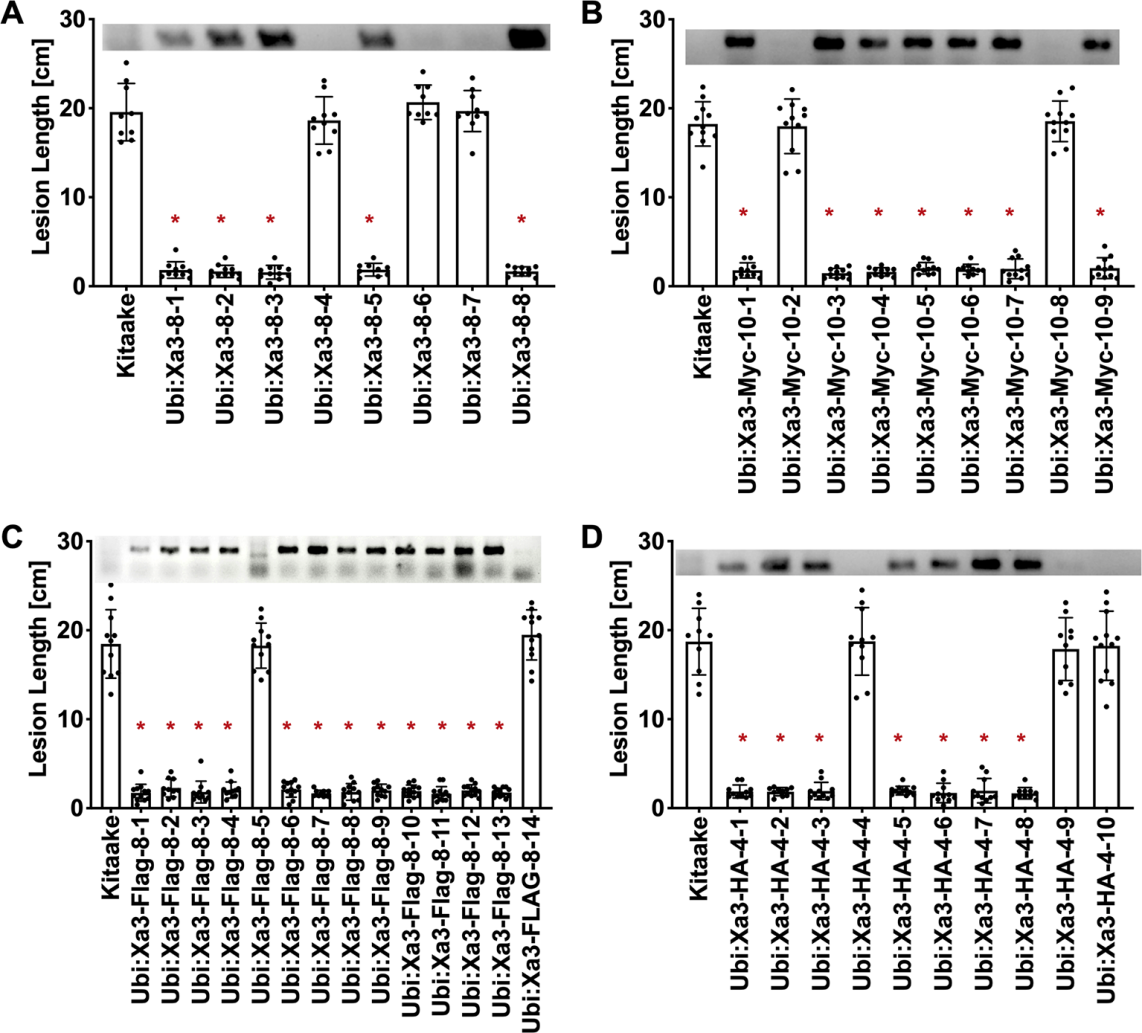
Resistance to *Xoo* strain PXO79 cosegregates with the *Xa3* transgene in the T1 progeny. Rice lines expressing *Xa3* are resistant to *Xoo* infection. The inoculation method was described in **Figure 1**. Bars represent means of 8–12 leaves. “*” indicates a statistically significant difference from Kitaake using Dunnett’s test (α = 0.05). The upper panel in each figure is genotyping data for the *Xa3* transgene by PCR using the forward primer annealing to the *Xa3* gene and the reverse primer annealing to the vector. **(A)** Lesion length data for eight *Ubi : Xa3* T1 plants. **(B)** Lesion length data for nine *Ubi : Xa3*:Myc T1 plants. **(C)** Lesion length data for fourteen *Ubi : Xa3:*Flag T1 plants. **(D)** Lesion length data for 10 *Ubi : Xa3*:HA T1 plants.

**Figure 3:**
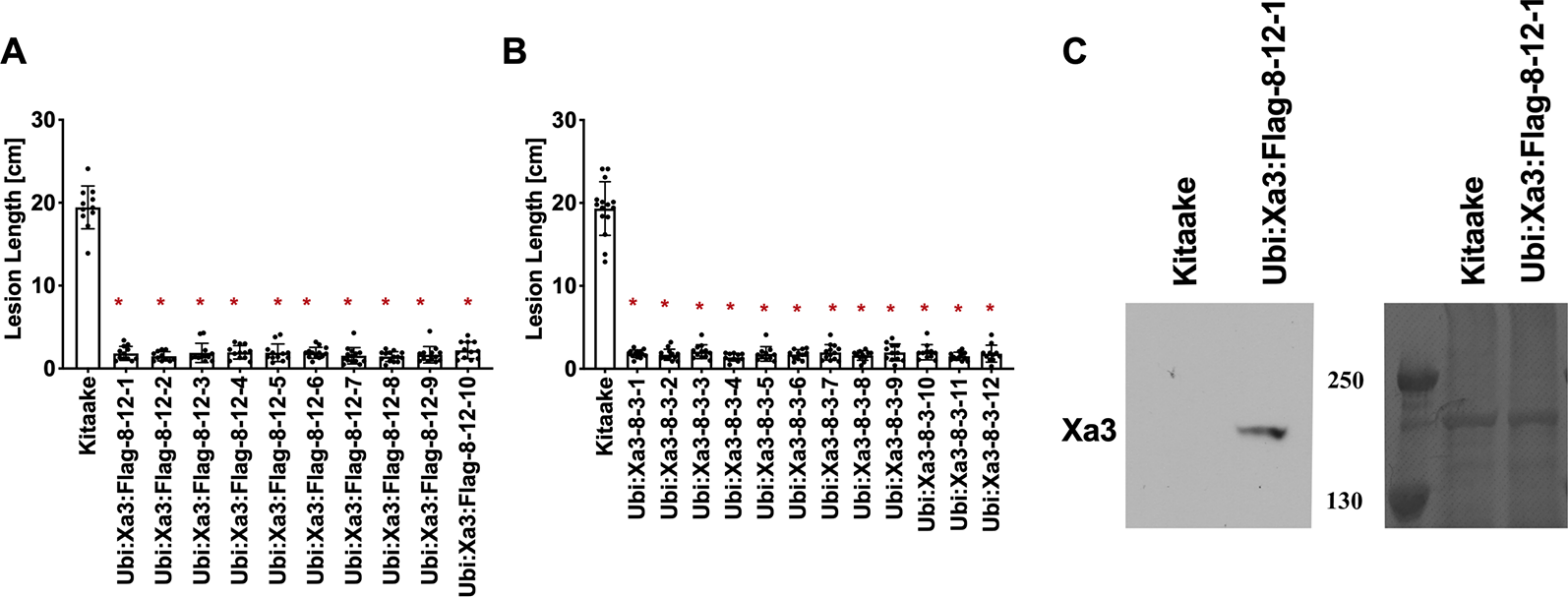
T2 lines derived from T1 parents *Ubi : Xa3-8-3* and *Ubi : Xa3*:Flag-*8-12* are resistant to PXO79. The inoculation method was described in **Figure 1**. Bars represent means of 8–12 leaves. “*” indicates statistically a significant difference from Kitaake using Dunnett's test (α = 0.05). **(A)** Lesion length data for 10 T2 plants of *Ubi : XA3:*Flag*-8-12*. **(B)** Lesion length data for twelve T2 plants of *Ubi : XA3-8-3*. **(C)** Western blot analysis of total protein extracted from five-week-old Kitaake and *Ubi : Xa3:*Flag*-8-12-1*. The experiment was repeated three times. The full-length XA3 was detected with an anti-Flag antibody (left). Approximately equal amounts of total proteins were loaded, as confirmed by Coomassie blue staining (right).

To assess if the resistance phenotype was transmitted to the next generation, T0 lines were self pollinated and T1 seeds were collected. These T1 plants together with rice plants lacking *Xa3* as controls were inoculated with *Xoo* strain PXO79 and assessed for resistance by measuring the lengths of disease-induced lesions (**Figure 2**). Inoculations were further carried out in *Ubi : Xa3:*Flag*-8-12* and *Ubi : Xa3-8-3* plants. We observed that T1 and T2 individuals that were PCR positive for the *Xa3* transgene (PCR targeting the *Xa3* sequence and *nos* terminator) co-segregated with resistance to PXO79 (**Figure 2**). Homozygous lines of *Ubi : Xa3:*Flag*-8-12* and *Ubi : Xa3-8-3* were used for subsequent experiments (**Figure 3**). We performed western blot assays to monitor the XA3:Flag protein in the *Ubi : Xa3:*Flag*-8-12-1* line using anti-Flag antibodies. Western blot analysis showed that the XA3:Flag protein is detectable in this line while Kitaake displays no detectable bands (**Figure 3C**). We did not detect the XA3:Myc or XA3:HA protein in western blot analyses.

### Infection Leads to Increased Expression of Defense-Related Genes in Detached Rice Leaves

**Figure 4:**
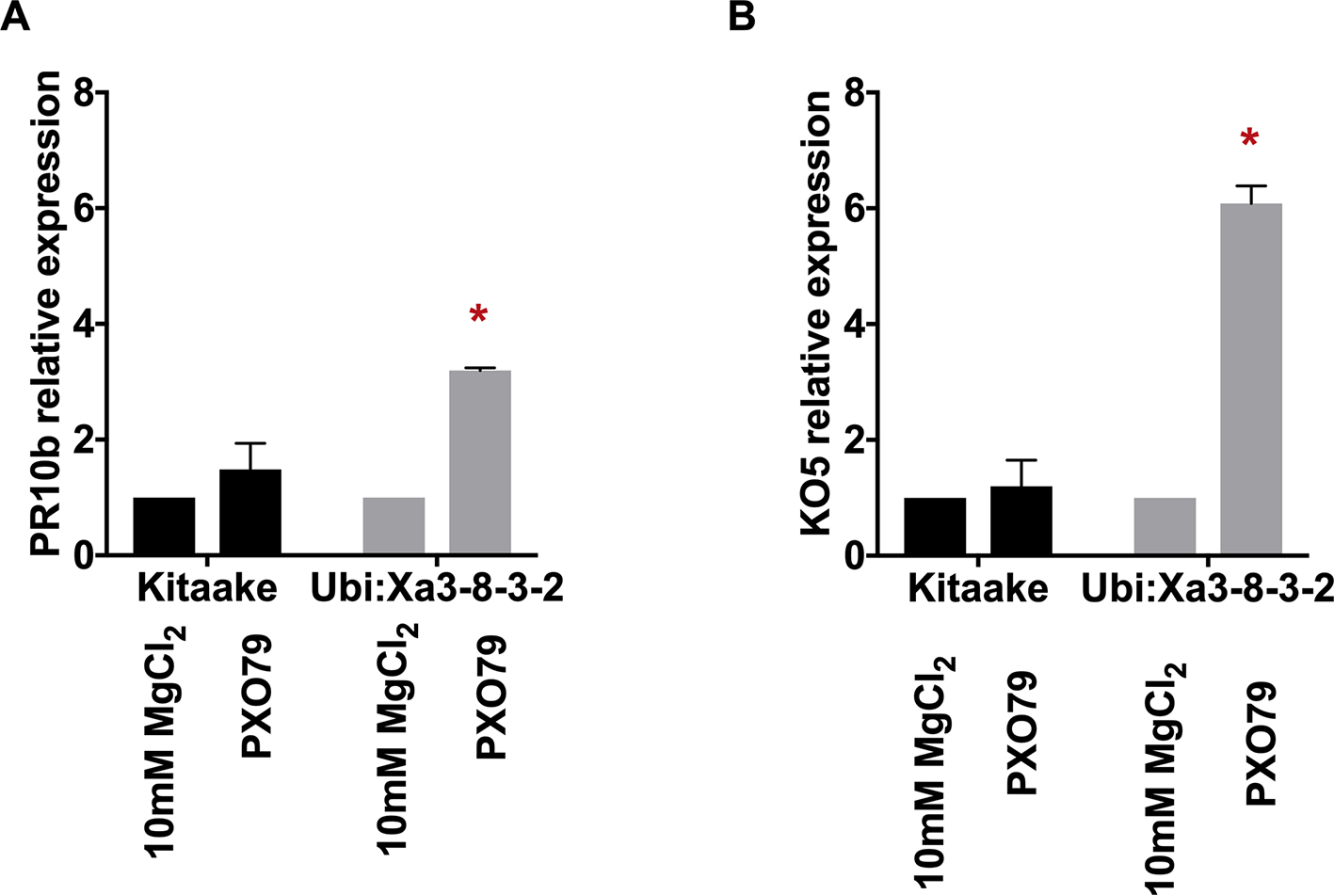
Marker genes are upregulated in detached rice leaves expressing XA3 (Ubi : Xa3-8-3-2) upon Xoo infection. The expression levels of marker genes *PR10b* (left) and *KO5* (right) are normalized to *actin* and compared to mock-treated samples. Bars indicate the mean expression level standard deviations of three biological replicates. “*” indicates a statistically significant difference using a T test (p < 0.05).

To assess the activation of the XA3-mediated immune response, we first set up a quick and reliable assay. Here we tested the induction of two genetic markers in detached rice leaves following PXO79 infection. We used rice leaves harvested from 4-week-old plants grown in a hydroponic system as described previously (Pruitt et al., 2015). Rice leaves were cut into 1-cm pieces and floated on 10 mM MgCl_2_ as mock treatment or 10 mM MgCl_2_ containing PXO79 cell suspensions (O.D. 600 = 0.1). The samples were kept under constant light and harvested at 24 h post-treatment for RNA extraction. Two previously described defense-related marker genes, *PR10b* (*LOC_Os12g36850*) and *KO5* (*LOC_Os06g37224*) were assayed as a readout for immune activation in *Xoo*-infected *Xa3* rice leaves (**Figure 4**) (Pruitt et al., 2015; Thomas et al., 2016). Gene expression changes were normalized to *actin* (*LOC_Os03g50885*) and compared to mock-treated samples. We observed that *PR10b* was upregulated 3.5 fold and *KO5* was elevated about 10 fold in the *Xa3* rice leaves treated with PXO79 compared to mock-treated samples. Neither was upregulated in the non-infected *Xa3* rice leaves or PXO79-infected Kitaake leaves. These results demonstrate that *PR10b* and *KO5* can be used as defense marker genes for XA3-mediated immunity.

### Neither *Xb24* Nor *Xb15* Overexpression Compromises the XA3-Mediated Immune Response

**Figure 5:**
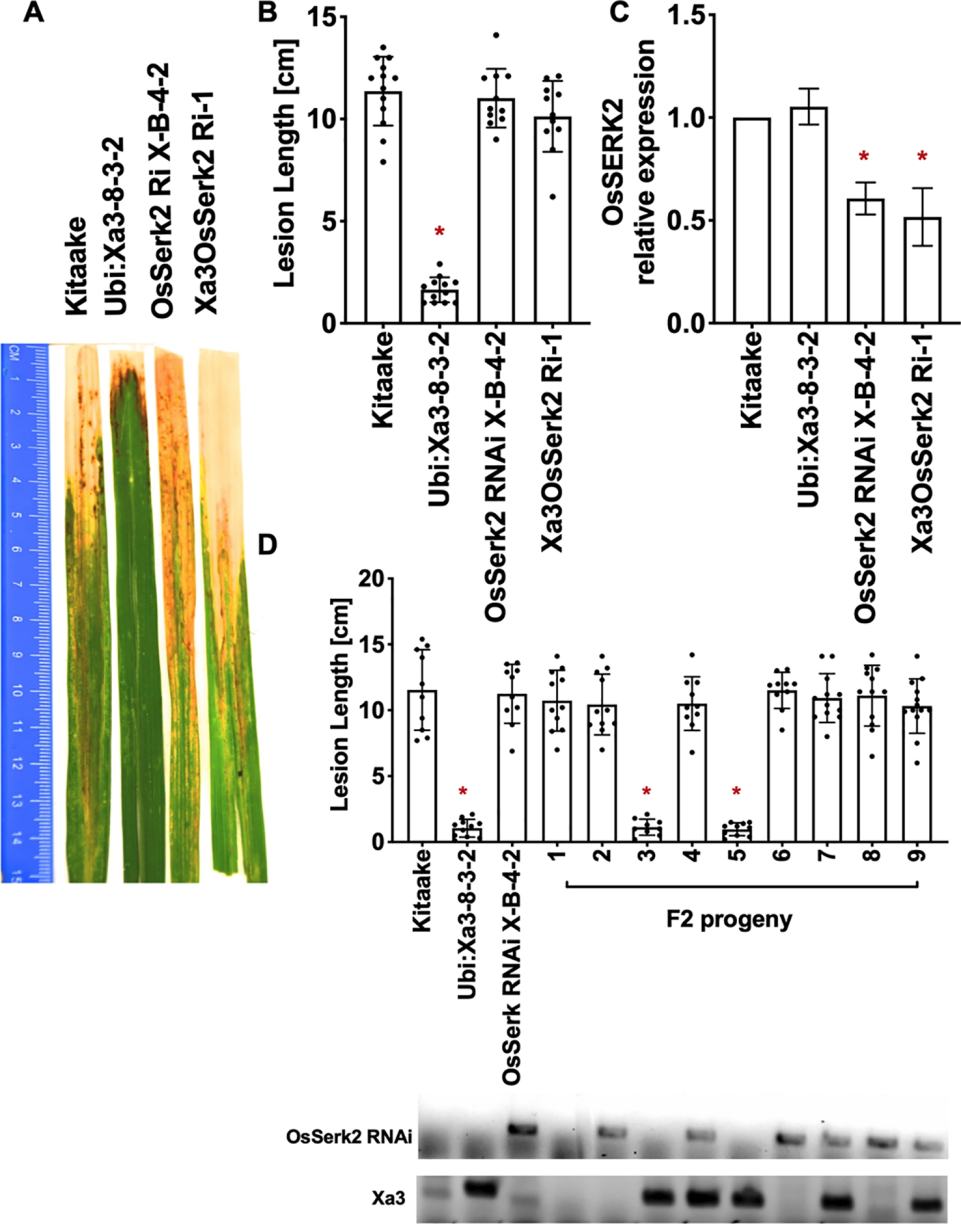
OsSERK2 is required for XA3-mediated immunity in the Kitaake genetic background. **(A)** Photographed 14 days after inoculation with *Xoo* strain PXO79. The inoculation method was described in **Figure 1**. Control lines used were Kitaake, *Ubi : Xa3-8-3-2* and *OsSerk2 RNAi X-B-4-2*. **(B)** The average lesion length data of the inoculated plants. Bars represent means of 8-12 leaves. “*” indicates a statistically significant difference from Kitaake using Dunnett's test (α = 0.05). **(C)** The qRT-PCR result shows that OsSERK2 is silenced in the inoculated F1 plant (*Xa3OsSerk2 Ri-1*). Gene expression measurement is the average of three biological replicates. “*” indicates a statistically significant difference from Kitaake using Dunnett's test (α = 0.05). **(D)** The average lesion length data of the inoculated F2 segregating population. Kitaake, *Ubi : Xa3-8-3-2* and *OsSerk2 RNAi X-B-4-2* were used as control lines. Bars represent means of 8–12 leaves. “*” indicates a statistically significant difference from Kitaake using Dunnett's test (α = 0.05). PCR genotyping for the *OsSerk* RNAi construct and *Xa3* transgene are shown below the bar graph.

**Figure 6:**
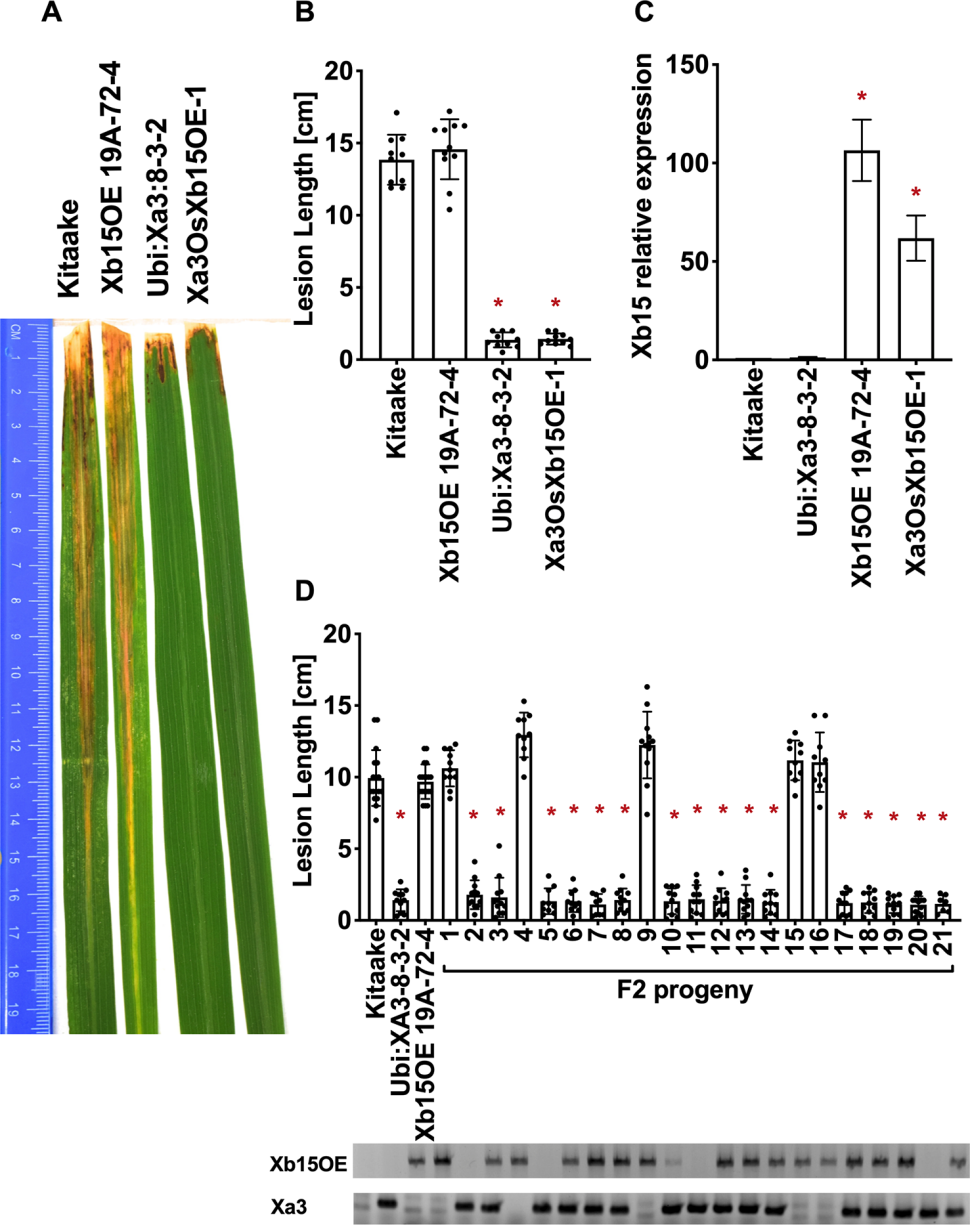
OsXB15 is not required for XA3-mediated immunity in the Kitaake genetic background. **(A)** Photographed 14 days after inoculation with *Xoo* strain PXO79. The inoculation method was described in **Figure 1**. Control lines used were Kitaake, *Ubi : Xa3-8-3-2* and *OsXb15OE 19A-72-4*. **(B)** The average lesion length data of the inoculated leaves (n = 8–12). “*” indicates a statistically significant difference from Kitaake using Dunnett's test (α = 0.05). **(C)** The qRT-PCR result shows that *OsXb15* is highly expressed in the inoculated F1 plant (*Xa3OsXb15OE-1*). Gene expression measurement is the average of three biological replicates. “*” indicates a statistically significant difference from Kitaake using Dunnett's test (α = 0.05). **(D)** The average lesion length data of the inoculated F2 segregating population. Kitaake, *Ubi : Xa3-8-3-2* and *OsXb15OE 19A-72-4* were used as control lines. Bars represent means of 8–12 leaves. “*” indicates a statistically significant difference from Kitaake using Dunnett's test (α = 0.05). PCR genotyping for the *OsXb15OE* construct and *Xa3* transgene are shown below the bar graph.

**Figure 7:**
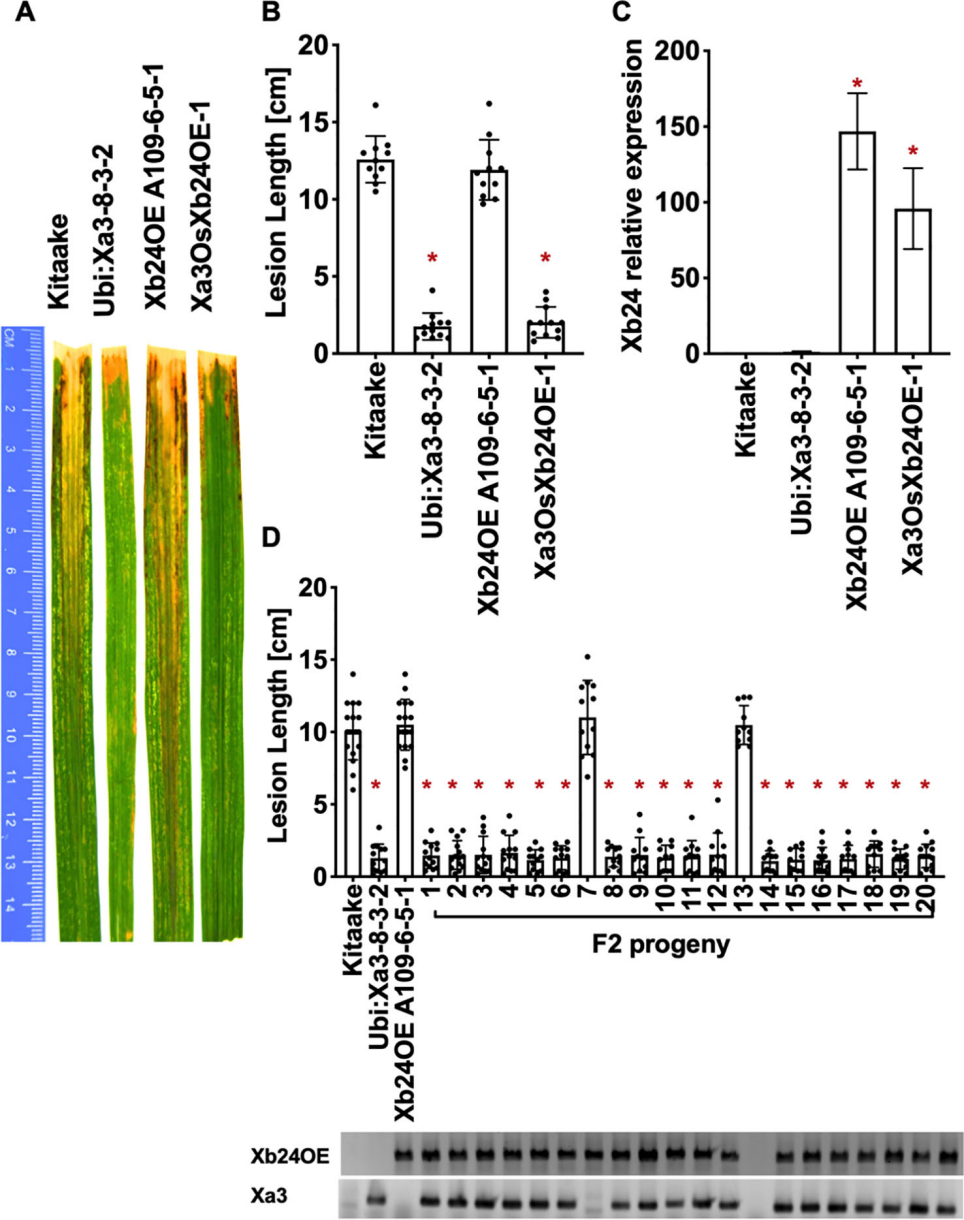
OsXB24 is not required for XA3-mediated immunity in the Kitaake genetic background. **(A)** Photographed 14 days after inoculation with *Xoo* strain PXO79. The inoculation method was described in **Figure 1**. Control lines used were Kitaake, *Ubi : Xa3-8-3-2* and *OsXb24OE A109-6-5-1*. **(B)** The average lesion length data of the inoculated leaves (n = 8–12). “*” indicates a statistically significant difference from Kitaake using Dunnett's test (α = 0.05). **(C)** The qRT-PCR result shows that *OsXb24* is highly expressed in the inoculated F1 plant (*Xa3OsXb24OE-1*). Relative gene expression measurement is represented as the average of three biological replicates. “*” indicates a statistically significant difference from Kitaake using Dunnett's test (α = 0.05). **(D)** The average lesion length data of the inoculated F2 segregating population. Kitaake, *Ubi : Xa3-8-3-2* and *OsXb24OE A109-6-5-1*were used as control lines. Bars represent mean lesion length on 8-12 leaves. “*” indicates a statistically significant difference from Kitaake using Dunnett's test (α = 0.05). PCR genotyping for the *OsXb24OE* construct and *Xa3* transgene are shown below the bar graph.

To test the role that XB24 or XB15 plays in the XA3-mediated immune response, we crossed *Xa3* Kitaake plants (*Ubi : Xa3-8-3-2* in Kitaake) with Kitaake lines overexpressing either *Xb24* (*Xb24OE* A109-6-5-1) or *Xb15* (*Xb15OE* 19A-72-4) individually (Park et al., 2008; Chen et al., 2010b). Overexpression of either of these genes in an XA21 background attenuates XA21-mediated resistance to *Xoo* (Park et al., 2008; Chen et al., 2010b). An *OsSerk2* RNAi line in Kitaake (*OsSerk2Ri* X-B-4-2) was used as a positive control (Chen et al., 2014). The F1 progeny were named *Xa3Xb24OE F1*, *Xa3Xb15OE F1,* and *Xa3OsSerk2Ri F1*. We isolated double-transgenic lines from independent crosses after PCR genotyping for each transgene and confirming *Xb15* or *Xb24* overexpression or *OsSerk2* silencing by qRT-PCR (**Figures 5C**, **6C** and **7C**). We next assessed the impact of *Xb24* and *Xb15* overexpression on *Xa3-*mediated immunity by inoculating with PXO79. Plants carrying *Xa3Xb15OE* and *Xa3Xb24OE* were as resistant as *Xa3* plants to PXO79, showing similar lesion lengths (**Figures 6A, B** and **7A, B**). Plants carrying both *Xa3* and *OsSerk2 RNAi* displayed much longer lesions at 14 dpi than *Xa3* control plants (Dunnett's test; α = 0.05) (**Figures 5A, B**). Inoculation of F2 segregating progeny confirmed these results (**Figure 5D**). The resistant phenotype co-segregated with the *Xa3* transgene. In the F2 progeny, the presence of *Xb24* and *Xb15* overexpression had no effects on XA3-mediated resistance (**Figures 6D** and **7D**). These results indicate that overexpression of XB24 and XB15 does not suppress the XA3-mediated immune response.

### The Intracellular Kinase Domains of XA3 and XA21 Differ

**Figure 8:**
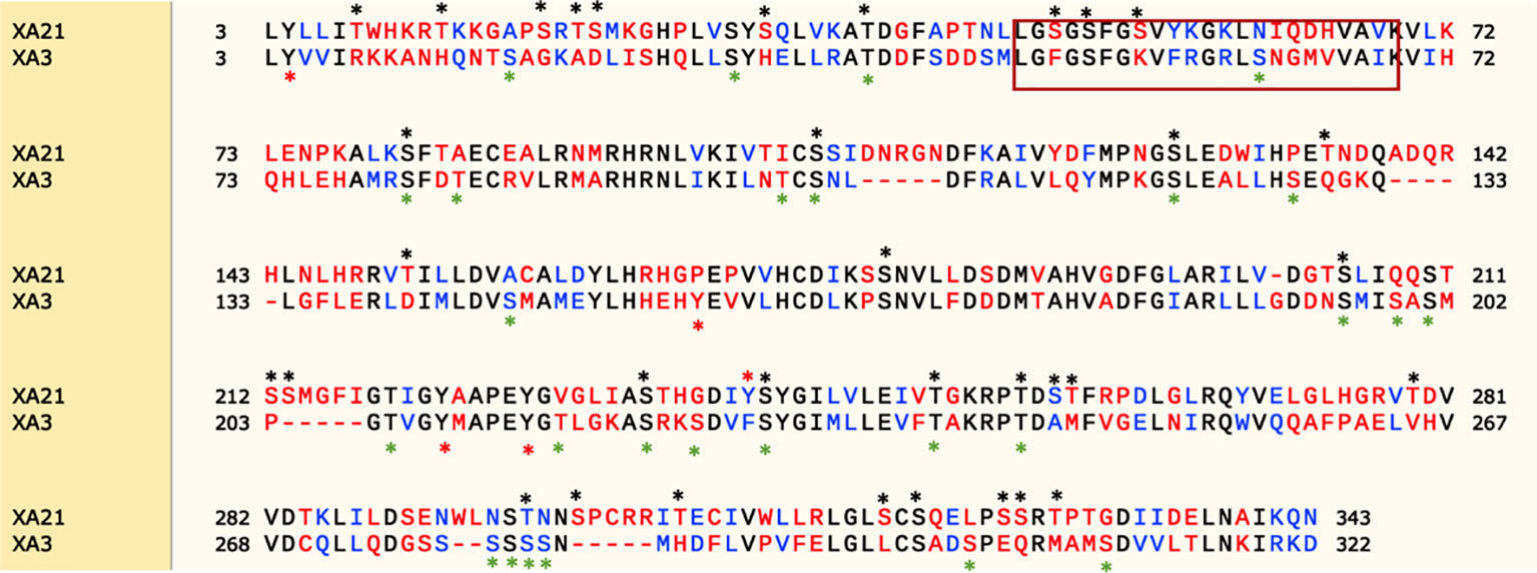
Protein sequence alignment of the XA21 and XA3 intracellular kinase domains. Snapgene (SnapGene software from GSL Biotech; available at snapgene.com) was used for sequence alignment and NetPhos 3.1 (Blom et al., 2004) was used to predict the phosphorylation sites. The red box represents the predicted ATP binding domain. Predicted serine/threonine or tyrosine phosphorylation sites are marked with a “*” above the sequence.

Protein kinases fall into two broad classes based on phosphorylation site specificity: serine/threonine-protein kinases and tyrosine-protein kinases (Hanks et al., 1988). XA21 encodes a serine/threonine protein kinase. There are 34 predicted serine/threonine and only 1 predicted tyrosine phosphorylation sites in the XA21 intracellular domain based on analysis by the phosphorylation sites prediction tool, NetPhos 3.1 (http://www.cbs.dtu.dk/services/NetPhos/) (**Figure 8**) (Blom et al., 2004). Previous studies demonstrated that Ser-686, Thr-688, Ser-689, and Thr-705, but none of the tested tyrosine residues, are required for XA21-mediated immunity (Xu et al., 2006; Chen et al., 2010a; Caddell et al., 2018).

We also used the NetPhos tool to identify 27 predicted Ser/Thr phosphorylation sites and 4 predicted tyrosine phosphorylation sites in the XA3 intracellular domain based on NetPhos3.1 (**Figure 8**). To date, there have been no reports experimentally validating the predicted XA3 phosphorylation sites.

Here we show that the amino acid sequence of the kinase domains of XA21 and XA3 are quite divergent. In particular, the amino acids predicted to be involved in ATP binding are not well conserved between the two receptor kinases (**Figure 8**). Based on domain analysis (https://pfam.xfam.org), the XA3 kinase domain is predicted to be a protein tyrosine kinase.

## Discussion

Previous studies showed that *Xa3* and *Xa21* confer resistance to *Xoo* when introgressed or expressed in diverse rice cultivars (Song et al., 1995; Sun et al., 2004). Here, we demonstrate that the rice gene *Xa3* also confers robust resistance to *Xoo* in the model rice cultivar Kitaake. The presence of Kitaake rice lines carrying *Xa3* and *Xa21* will facilitate further research to investigate other components that may regulate both XA3- and XA21-mediated immunity in the same rice genetic background. Genetic analysis results show that overexpression of two previously characterized negative regulators of *Xa21* signaling, *Xb24* and *Xb15* (Park et al., 2008; Chen et al., 2010b), had no effects on XA3-mediated immunity in Kitaake. To validate this result, additional experiments are needed. For example, evaluation of Kitaake lines silenced or knocked out for *Xb15* or *Xb24* would reveal if these genes could serve as positive regulators of XA3-mediated immunity. Because XB15 and XB24 are both members of multi-gene families, it is not possible to rule out a role for other family members in modulating the immune response. For example, XB15 is one of 76 serine/threonine type 2C protein phosphatases (PP2C) in rice and XB15 shows high similarity with several rice PP2C members, with the PP2C encoded by *Os03g25600* being the closest homolog (Park et al., 2008; Yang et al., 2014).

Plant PRRs mostly belong to Ser/Thr kinases (Shiu and Bleecker, 2001), and Tyr phosphorylation of receptor kinases have been mostly reported in plant RD kinases signaling such as for the receptor kinases BIK1 (*Botrytis*-induced kinase 1) and BAK1 (brassinosteroid insensitive 1-associated kinase 1) which are involved in plants growth and immunity (Lin et al., 2014), and the Tyr^428^ phosphorylation of CERK1 (chitin elicitor receptor kinase 1) is required for fungal chitin triggered immune signaling (Liu et al., 2018). For the non-RD kinases, the Arabidopsis receptor kinase EF-TU Receptor (EFR), has been reported being activated upon ligand binding by phosphorylation on its tyrosine residues (Macho et al., 2014). Here, we also show that the amino acid sequence of the kinase domains of XA21 and XA3 are quite divergent, and that XA3 carries fewer predicted Ser/Thr phosphorylation sites compared with XA21. The XA3 kinase domain is predicted to be a protein tyrosine kinase. In addition, the amino acids predicted to be involved in ATP binding are not well-conserved between the two kinase domains. These observed differences in the XA21 vs. XA3 kinase amino acid sequences may dictate different requirements for binding with downstream signaling components.

## Data Availability Statement

All datasets generated for this study are included in the article/supplementary material.

## Author Contributions

FL and WZ performed the experiments. TW, BS and PR contributed to conception. FL and PR wrote the manuscript. All authors edited the manuscript and read and approved the submitted version.

## Funding

Supported by NIH GM59962 and GM122968 to PR.

## Conflict of Interest

The authors declare that the research was conducted in the absence of any commercial or financial relationships that could be construed as a potential conflict of interest.
